# 超高效液相色谱-静电场轨道阱高分辨质谱法测定硝苯地平中痕量基因毒性杂质

**DOI:** 10.3724/SP.J.1123.2021.06008

**Published:** 2022-03-08

**Authors:** Changchuan GUO, Huijie TAN, Qi LIU, Tengfei GONG, Xue WANG, Chenglin WANG, Yuwen XU

**Affiliations:** 1.山东省食品药品检验研究院, 国家药品监督管理局仿制药研究与评价重点实验室, 山东省仿制药一致性评价工程技术研究中心, 山东 济南 250101; 1. Shandong Institute for Food and Drug Control, National Medical Products Administration (NMPA) Key Laboratory for Research and Evaluation of Generic Drugs, Shandong Research Center of Engineering and Technology for Consistency Evaluation of Generic Drugs, Jinan 250101, China; 2.威海市食品药品检验检测中心, 山东 威海 264299; 2. Weihai Institute for Food and Drug Control, Weihai 264299, China; 3.山东大学齐鲁医学院药学院, 山东 济南 250012; 3. School of Pharmacy, Cheeloo College of Medicine, Shandong University, Jinan 250012, China

**Keywords:** 超高效液相色谱, 高分辨质谱, 基因毒性, 硝苯地平, 杂质, ultra high performance liquid chromatography (UHPLC), high resolution mass spectrometry (HRMS), genotoxicity, nifedipine, impurities

## Abstract

建立了测定硝苯地平中基因毒性杂质2、6和12的超高效液相色谱-静电场轨道阱高分辨质谱法(UHPLC-Orbitrap HRMS)。样品以甲醇为溶剂,提取后直接进样分析。采用ACE EXCEL^TM^ 3 C18-AR色谱柱(150 mm×4.6 mm, 3 μm)分离,流动相为甲醇-0.1%甲酸水(65:35, v/v),等度洗脱。质谱部分采用电喷雾电离(ESI)源。采用正离子平行反应监测(PRM)扫描模式,质谱分辨率为35000 FWHM,杂质2、6、12的[M+H]^+^母离子准确质量数分别为*m/z* 347.1230、361.1026、347.1230,提取[M+H]^+^碎片离子准确质量数分别为*m/z* 315.0968、298.1069、315.0968,归一化碰撞能量(NCE)分别为10%、42%、10%,外标法定量。对方法进行了详细的方法学验证,结果表明,该法专属性良好,溶剂对杂质测定无干扰;杂质2、6、12质量浓度与其峰面积在0.2~100 ng/mL范围内呈现良好的线性关系,相关系数(*r*)均≥0.9998;杂质2、6、12在低、中、高3个水平下的回收率为96.9%~105.0%, RSD为1.21%~5.12%,检出限均为0.05 ng/mL,定量限均为0.2 ng/mL。应用该方法对3批硝苯地平样品中的杂质2、6、12进行测定,3批样品均未检出杂质6,但均检出杂质2和杂质12,其检出量未超过限度。该方法灵敏、快速、准确,操作简便,可为药企对硝苯地平的质量控制提供参考,并为药监部门的监管提供有力的技术支持。

## Abstract (en)

A method based on ultra high performance liquid chromatography-electrostatic field orbitrap high resolution mass spectrometry (UHPLC-Orbitrap HRMS) was established for the determination of genotoxic impurities 2, 6, and 12 in nifedipine. After extraction with methanol, the sample was injected into the UHPLC-Orbitrap HRMS system for analysis. An ACE EXCEL^TM^ 3 C18-AR column (150 mm×4.6 mm, 3 μm) was used for chromatographic separation. The mobile phase was methanol-0.1% formic acid aqueous solution (65:35, v/v). The flow rate was 0.6 mL/min, while the column temperature and autosampler temperature were set as 35 ℃ and 8 ℃, respectively. The divert valve switching technique was used to protect the mass spectrometer. The six-way valve was set to divert the eluent of 7.5-11.6 min to waste and the rest of the eluent into the mass spectrometer. The Orbitrap mass spectrometer was coupled with the UHPLC system by an electrospray ion (ESI) source. The sheath gas and auxiliary gas flow rates were 60 and 20 arb (arbitrary units), respectively. The spray voltage was 3.5 kV, while the capillary temperature and auxiliary gas heater temperature were set as 350 ℃ and 400 ℃, respectively. The positive ion parallel reaction monitoring (PRM) scanning mode was adopted, and the mass spectral resolution was set to 35000 FWHM. The accurate masses of the [M+H]^+^ precursor ions of impurities 2, 6, and 12 were *m/z* 347.1230, 361.1026, and 347.1230, respectively. The accurate masses of the extracted [M+H]^+^ fragment ions of impurities 2, 6, and 12 were *m/z* 315.0968, 298.1069, and 315.0968, respectively. The normalized collision energies (NCEs) were optimized to 10%, 42%, and 10% for impurities 2, 6, and 12, respectively. The external standard method was utilized for quantitative analysis. The established method was validated in detail by investigating the specificity, linear range, limit of detection (LOD), limit of quantification (LOQ), recovery, precision, and stability. This method had good specificity, and the solvent did not interfere with the determination of impurities. The peak areas of impurities 2, 6, and 12 as well as their concentrations showed good linear relationships in the ranges of 0.2-100 ng/mL, with all correlation coefficients (*r*)≥0.9998. The recoveries of impurities 2, 6, and 12 at three levels (low, medium, and high) were in the range of 96.9%-105.0%, while the RSDs were between 1.21% and 5.12%. The LODs were 0.05 ng/mL and the LOQs were 0.2 ng/mL for all three impurities. This analytical method was used to determine impurities 2, 6, and 12 in three batches of nifedipine samples. Impurity 6 was not detected in the three batches, but impurities 2 and 12 were detected in all the three samples, and the detection amount was within the limit. The developed method is sensitive, fast, accurate, and easy to operate. It can provide a reference for the quality control of nifedipine by pharmaceutical companies and extend strong technical support for the supervision by drug regulatory authorities.

二氢吡啶类钙拮抗剂是一类降压作用强、临床应用广、上市品种多的心血管疾病治疗药^[1,2]^,主要包括硝苯地平、氨氯地平、乐卡地平、尼莫地平、尼卡地平、尼群地平、非洛地平、拉西地平等在临床中常用的药物。硝苯地平是一种常见的第一代二氢吡啶类钙拮抗剂,可选择性地抑制钙离子进入心肌细胞和平滑肌细胞的跨膜转运,抑制钙离子从细胞内释放,且不改变血浆中钙离子浓度,对心血管平滑肌钙通道有阻滞作用,干扰肌肉的兴奋收缩,有较强的扩张血管作用,临床上主要用于高血压、心绞痛、心律失常及其他心血管疾病的治疗^[3,4]^。 当前,对于硝苯地平杂质的研究主要集中在非致突变性杂质^[5,6,7,8,9,10]^,而对其基因毒性杂质的调查鲜有报道。硝苯地平杂质2、6、12的化学结构如图1所示,杂质2和杂质12含有的硝基基团、杂质6含有的氮杂芳基*N*-氧基团均属于基因毒性警示结构,应按照致突变性杂质的要求制定杂质限值^[11]^。

**图1:**
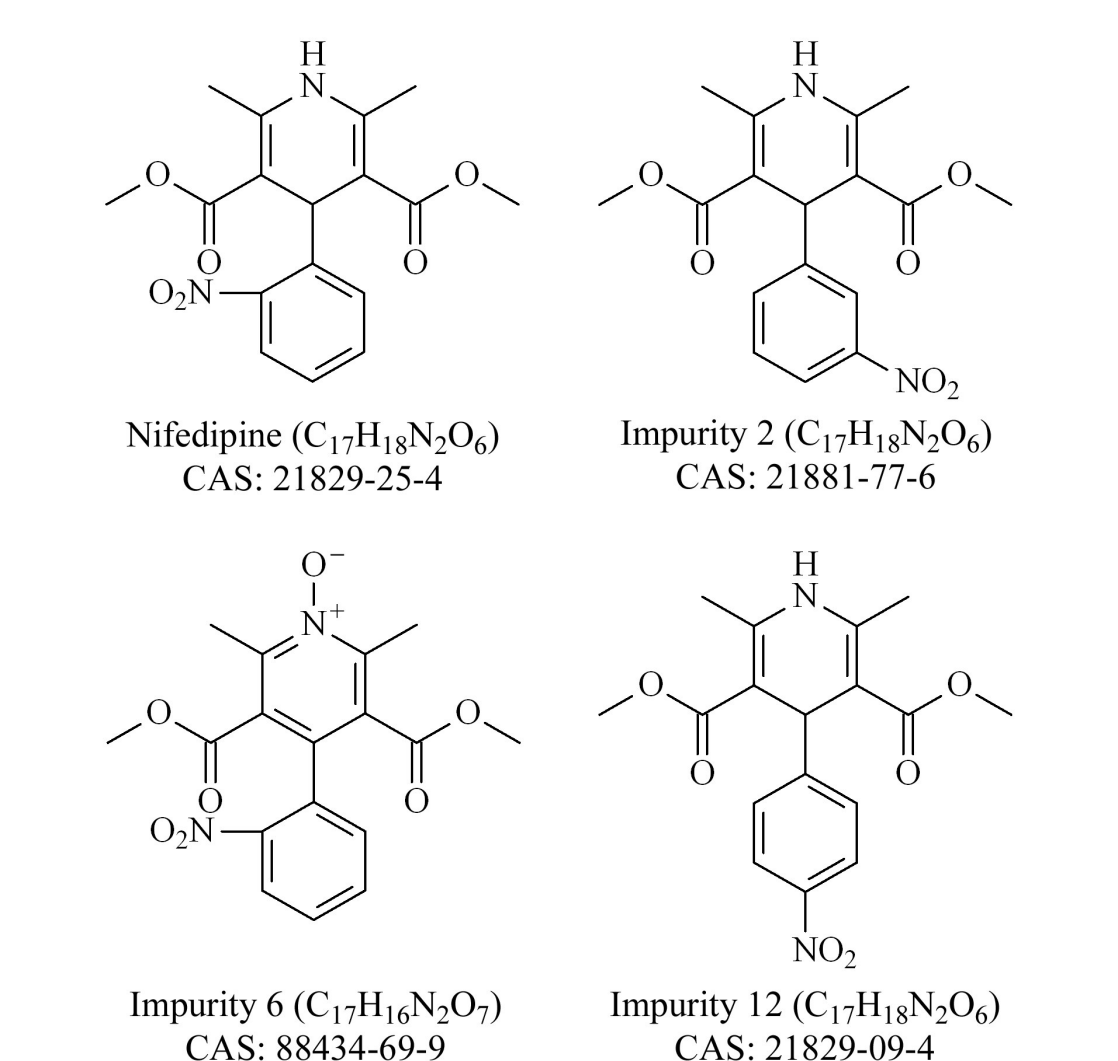
硝苯地平及杂质2、6和12的结构式

目前已报道的硝苯地平杂质的测定方法主要有高效液相色谱法(HPLC)^[5,7,8]^、超高效液相色谱法(UHPLC)^[10]^和液相色谱-质谱法(LC-MS)^[6]^。近年来,静电场轨道阱(Orbitrap)高分辨质谱技术凭借其超高质量精度、高灵敏度和高选择性的优势,在药品杂质分析方面的应用逐渐增多^[12,13,14,15]^。本研究建立了UHPLC-Orbitrap HRMS测定硝苯地平原料药中基因毒性杂质2、6和12的方法,并进行了详细的方法学验证。

## 1 实验部分

### 1.1 仪器、试剂与材料

Thermo Q Exactive Plus^TM^超高效液相色谱-高分辨质谱联用系统包括Ultimate 3000三元泵、自动进样器、柱温箱、DAD检测器以及Orbitrap高分辨质谱部分,Tune2.9软件用于质谱仪的调谐和质量轴的校正,XCalibur^TM^ 4.0软件用于设置仪器方法、编辑序列和处理数据,以上均购于Thermo公司(德国); Mettler XS205型电子天平(Mettler Toledo公司,瑞士)用于精密称量。

硝苯地平原料药由山东省某医药公司提供;杂质2对照品购自STD公司(美国),批号为196132N-SL-01,纯度为98.7%;杂质6对照品购自QCC公司(美国),批号为29-APR-19-32,纯度为99.98%;杂质12对照品购自STD公司(美国),批号为1656312B-SL-01,纯度为99.6%;质谱级甲醇(纯度99.9%)、甲酸(纯度>99%)均购自Fisher公司(美国);实验用18.2 MΩ·cm纯水由Milli-Q Advantage A10超纯水系统(Millipore公司,美国)制得。

### 1.2 实验条件

1.2.1 对照品溶液的制备

分别精密称取杂质2、6和12对照品10.07、10.20和10.26 mg,分别以甲醇作为稀释剂,制成质量浓度分别为397.56、407.92和408.76 μg/mL的对照品储备液。精密移取上述对照品储备液适量,加甲醇稀释得质量浓度约为20 μg/mL的单一对照品溶液。精密移取20 μg/mL各单一对照品溶液适量,加甲醇逐步稀释,得到质量浓度约为0.05、0.2、0.5、2、5、10、20、50、100 ng/mL的杂质2、6、12混合对照品标准溶液。

1.2.2 样品前处理

取硝苯地平约25 mg,精密称定,置于50 mL离心管中,精密加入甲醇25 mL,涡旋使其完全溶解,摇匀即得。

1.2.3 色谱条件

色谱柱为ACE EXCEL^TM^ 3 C18-AR柱(150 mm×4.6 mm, 3 μm),柱温为35 ℃,自动进样器温度为8 ℃,流动相为甲醇-0.1%甲酸水溶液(65:35, v/v),等度洗脱,流速为0.6 mL/min,分析时间为18 min,进样量为5 μL,检测波长为235 nm。

六通阀切换设置:保留时间为7.5~11.6 min时,流动相进入废液,其余保留时间,流动相进入质谱检测。

1.2.4 质谱条件

电喷雾电离源,正离子模式,喷雾电压3.5 kV;毛细管温度350 ℃,辅助气加热器温度400 ℃;鞘气流量60 arb,辅助气流量20 arb。采用平行反应监测(PRM)质谱扫描模式,分辨率设为35000 FWHM,隔离窗口(isolation window)设为*m/z* 1.5,微扫描(microscans)设为3,自动增益控制(AGC target)设为2×10^5^,最大离子注入时间(maximum IT)设为100 ms。杂质2、6、12的具体质谱参数见表1。

**表1:** 杂质2、6、12的质谱参数

Impurity	Scan time/min	Precursor ion (m/z)	NCE/%	Product ion (m/z)
Impurity 2	13.0-18.0	347.1230	10	315.0968
Impurity 6	0-18.0	361.1026	42	298.1069
Impurity 12	13.0-18.0	347.1230	10	315.0968

NCE: normalized collision energy.

## 2 结果和讨论

### 2.1 实验条件考察

2.1.1 质谱条件优化

目前尚无硝苯地平杂质2、6、12的毒理学数据,可采用ICH M7 R1推荐的毒理学关注阈值(TTC)来计算可接受摄入量,即单个杂质的可接受摄入量为1.5 μg/d^[16]^。硝苯地平每日最大用量为80 mg,故硝苯地平中杂质2、6、12限值的计算公式^[11]^为杂质限值=杂质可接受摄入量÷药物每日最大用量=18.75 ng/mg。此限值比硝苯地平有关物质的限度(0.1%或0.2%)^[11]^低2个数量级,对仪器灵敏度要求较高,宜采用LC-MS进行测定。

本研究所使用的Thermo Q Exactive Plus^TM^超高效液相色谱-高分辨质谱联用系统的质谱部分串联了四极杆和Orbitrap两个质量分析器,拥有多样化的质谱扫描模式。在开发质谱方法时,考察了目标物选择性离子监测(Targeted SIM)、全扫描-数据依赖的二级扫描(Full MS/dd MS^2^)、PRM等不同的质谱扫描模式,结果发现在杂质2、6、12质量浓度≥10 ng/mL时,3种扫描模式灵敏度相当,而当杂质2、6、12质量浓度低于10 ng/mL时,在Targeted SIM或Full MS/dd MS^2^模式下采集的总离子流色谱图中无法提取杂质的色谱峰,但PRM模式下杂质2、6、12质量浓度为0.05~10 ng/mL时依然能够获得较好的提取离子色谱图。因此,本研究采用PRM质谱扫描模式。采用Tune2.9软件优化杂质2、6、12的碰撞能量,选择合适的提取碎片离子,并对离子源气流速、温度、电压进行了精细调节和逐级优化,实现了杂质2、6、12的痕量检测,最佳质谱条件如1.2.4节所示,3种杂质的高分辨二级质谱图及碎片离子预测结构式如图2所示。

**图2:**
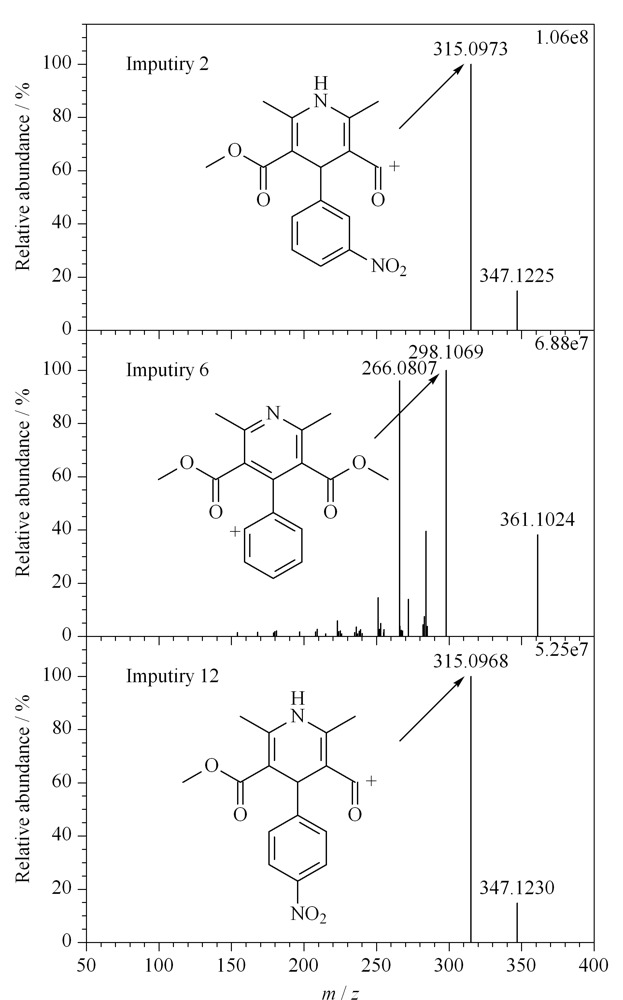
杂质2、6、12的二级质谱图及碎片离子结构预测

2.1.2 色谱条件的优化

在本研究中,杂质2和杂质12与硝苯地平为同分异构体,3者分子式、准确质量数、提取离子质量数均完全相同,因此杂质2、12及硝苯地平色谱峰必须达到基线分离才能够进行准确测定。另一方面,由于供试品溶液中含有高质量浓度的药品主成分硝苯地平(约1.0 mg/mL),如果高浓度硝苯地平进入质谱系统,会污染离子源并产生严重的基质效应,影响痕量杂质2、6、12的准确测定。因此,应充分优化色谱条件,尽可能提高杂质2、6、12与硝苯地平的色谱分离度,再通过在线六通阀将硝苯地平色谱峰切换到废液,避免高浓度硝苯地平进入质谱系统。实验考察了岛津Shim-pack GIST^TM^苯基-己基柱(150 mm×4.6 mm, 3 μm)和ACE EXCEL^TM^ 3 C18-AR柱(150 mm×4.6 mm, 3 μm)等两种不同类型色谱柱的分离效果。当采用岛津Shim-pack GIST^TM^苯基-己基柱时,杂质2与硝苯地平色谱峰保留时间相隔太近(<1 min),阀切换很难将硝苯地平主成分彻底切换,对其准确测定造成影响。采用ACE EXCEL^TM^ 3 C18-AR色谱柱(150 mm×4.6 mm, 3 μm)时,杂质6、硝苯地平、杂质2、杂质12的保留时间分别为6.25、10.74、14.26和15.91 min。硝苯地平与3种杂质均实现了良好的色谱分离,通过六通阀控制将保留时间为7.5~11.6 min时的流动相切入废液,有效避免了高浓度硝苯地平的基质干扰,保护了质谱离子源,降低了杂质2和杂质12的基线噪音,使杂质测定更加准确可靠。

2.1.3 样品前处理方法优化

中国药典载明:硝苯地平在丙酮或三氯甲烷中易溶,在乙醇中略溶,在水中几乎不溶^[11]^。然而,丙酮和三氯甲烷溶解性太强,会对液相色谱柱和液相色谱管路造成严重影响,同时也会使色谱图上出现一些干扰峰。此外,考虑到甲醇与流动相的兼容性明显优于乙醇。药典载明,硝苯地平以甲醇作为溶剂可配制成质量浓度为1 mg/mL的溶液^[11]^。因此,综合以上考虑,本研究选用甲醇作为提取溶剂,硝苯地平样品含量定为1 mg/mL。结果表明,硝苯地平与杂质2、6、12均能完全溶于甲醇,形成澄清透明的溶液,前处理简单。

### 2.2 方法学考察

2.2.1 专属性

将甲醇溶剂和杂质2、6、12混合对照品溶液分别进样测定,记录色谱图。甲醇溶剂的图谱如图3a所示,通过与对照品溶液提取离子色谱图(见图3b)对比可知,杂质2、6、12相同保留时间处无干扰峰存在,表明本方法专属性良好。

**图3:**
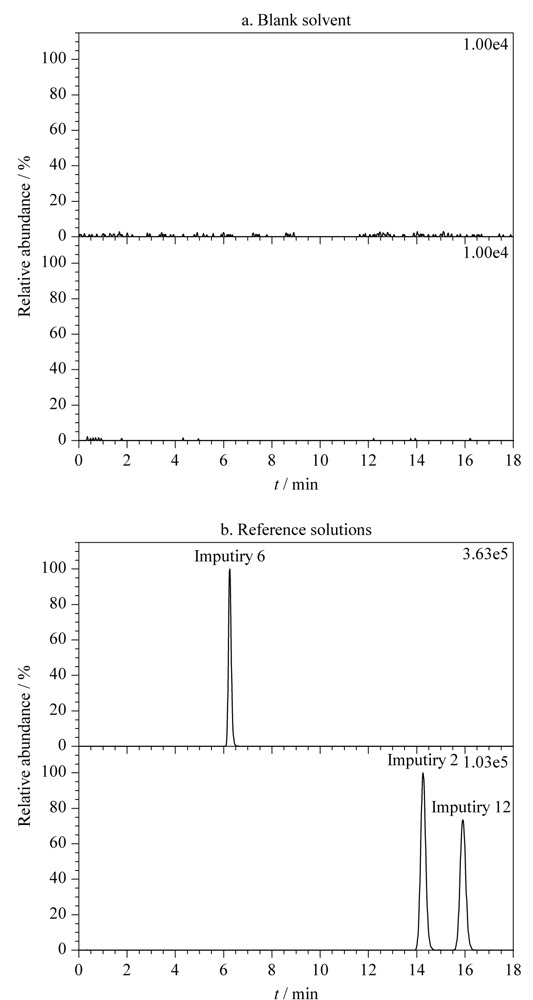
(a)空白溶剂及(b)对照品溶液的提取离子色谱图

2.2.2 线性范围

分别取0.2、0.5、2、5、10、20、50、100 ng/mL的杂质2、6、12混合对照品溶液进样测定,以峰面积作为*y*、分析物质量浓度(ng/mL)作为*x*,不加权重拟合线性校正曲线,建立回归方程,计算相关系数(*r*),如表2所示。结果表明,杂质2、6、12峰面积与其质量浓度分别在0.2~100 ng/mL范围内呈现良好的线性关系,*r*均≥0.9998。

**表2:** 杂质2、6、12的线性方程和相关系数

Analyte	Linear equation	r
Impurity 6	y=6.71×10^5^x-3.68×10^4^	0.9999
Impurity 2	y=3.38×10^5^x-1.81×10^4^	0.9998
Impurity 12	y=2.84×10^5^x-2.29×10^4^	0.9999

*y*: peak area; *x*: mass concentration, ng/mL.

2.2.3 检出限和定量限

精密量取质量浓度为0.2 ng/mL的杂质2、6、12混合对照品溶液,用甲醇逐级稀释,按照信噪比(*S/N*)≥3和≥10分别计算检出限和定量限。结果表明,3种杂质的检出限均为0.05 ng/mL(即0.05 ng/mg),定量限均为0.20 ng/mL(即0.20 ng/mg)。3次定量限平行试验中杂质2、6、12的平均信噪比分别为23、21和17,峰面积RSD分别为5.4%、4.9%和6.0%。定量限提取离子色谱图如图4所示。

**图4:**
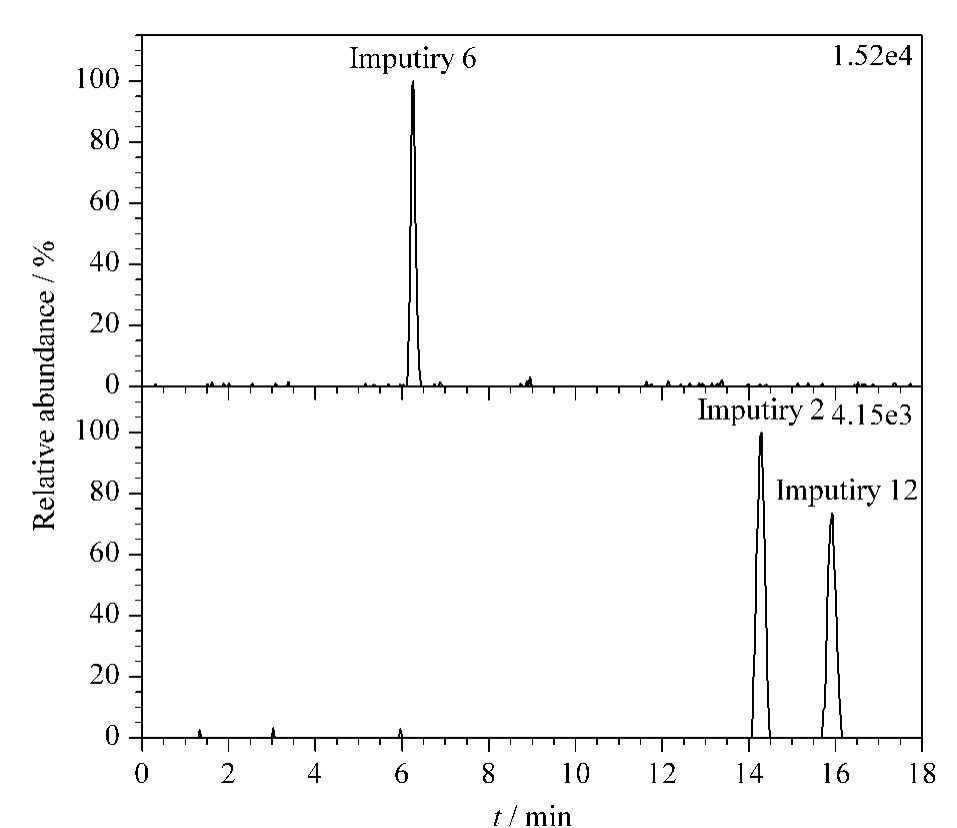
定量限下杂质2、6和12的提取离子色谱图

2.2.4 回收率和精密度

取硝苯地平约25 mg(批号1911231),精密称取9份(低、中、高水平各3份),先分别精密加入适量的杂质2、6、12混合对照品溶液,后续操作按照1.2.2节描述处理,得到加标回收试验用供试品溶液,进样测定。结果如表3所示,杂质2、6、12的加标回收率为96.9%~105.0%, RSD为1.21%~5.12%,符合药典规定,表明杂质2、6和12回收率和精密度良好。

**表3:** 硝苯地平中杂质2、6、12的加标回收率(*n*=3)

Analyte	Background/ (ng/mL)		Spiked level/ (ng/mL)	Measured/ (ng/mL)	Recovery/ %	RSD/ %
Impurity 2		0.321	0.204	0.547	104.2	5.12
			0.409	0.747	102.3	1.85
			0.613	0.922	98.7	3.44
Impurity 6		nd	0.199	0.209	105.0	4.33
			0.398	0.387	97.2	3.76
			0.596	0.605	101.5	2.91
Impurity 12		0.402	0.204	0.587	96.9	4.97
			0.408	0.843	104.1	1.21
			0.612	1.031	101.7	2.58

nd: not detected.

2.2.5 稳定性

将质量浓度约为0.20 ng/mL的杂质2、6、12混合对照品溶液保存于进样瓶中,在自动进样器中按照实验条件放置0、8、12、24 h后进样测定,杂质2、6、12峰面积的RSD值分别为4.3%、5.1%和4.5%,说明该条件下杂质2、6、12在24 h内稳定性良好。

2.2.6 耐用性

微调分析条件,将柱温分别调整为32 ℃和38 ℃、流速分别调整为0.55 mL/min和0.65 mL/min时,杂质的色谱峰形无变化,色谱保留时间和峰面积发生微小改变,各杂质峰面积和保留时间RSD值均<10%。因此,测定条件轻微变动不影响杂质2、6、12的检测,方法耐用性良好。

### 2.3 实际样品检测

采用已验证的方法,对3批硝苯地平原料处理后,进样测定,外标法计算样品中杂质2、6、12含量,结果见表4,代表性提取离子色谱图如图5所示。3批样品均未检出杂质6,但均检出杂质2和杂质12,检出含量分别为0.315~0.382 ng/mg和0.285~0.404 ng/mg,远低于限度规定(18.75 ng/mg)。因此,样品中杂质2、6、12的检出量符合规定。

**表4:** 硝苯地平样品检测结果

Batch number	Impurity	Content/(ng/mg)
1911211	2	0.315
	6	nd
	12	0.285
1911231	2	0.321
	6	nd
	12	0.402
1910281	2	0.382
	6	nd
	12	0.404

**图5:**
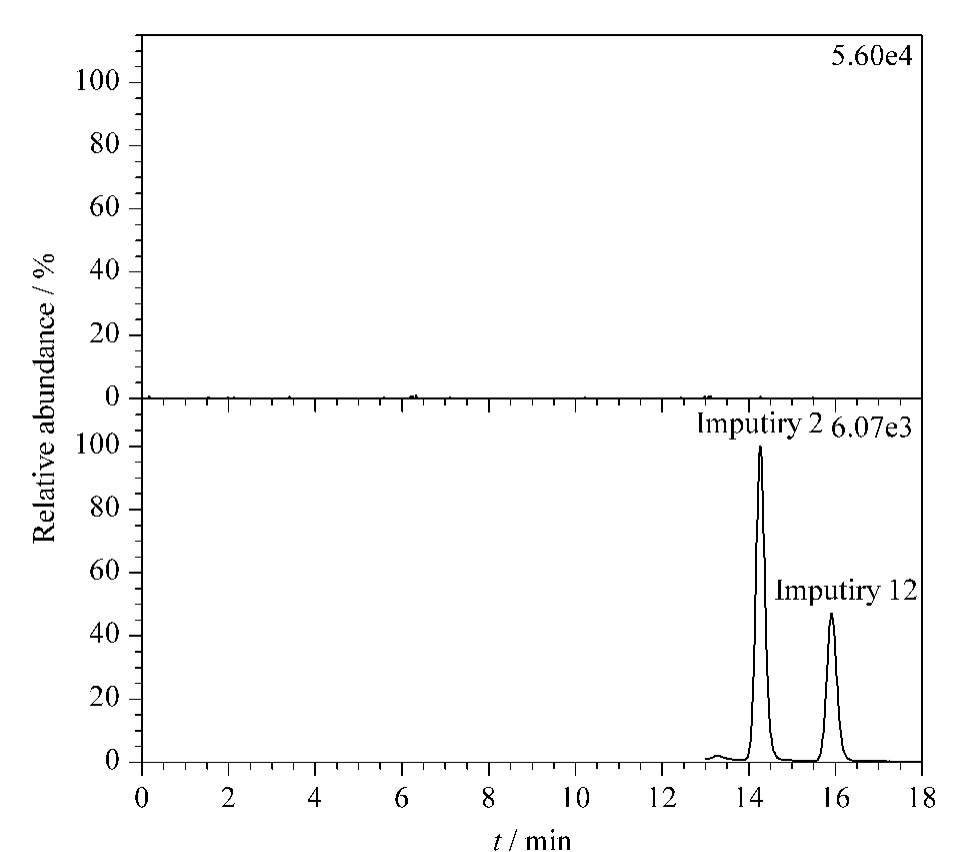
供试品提取离子色谱图

## 3 结论

本研究建立了硝苯地平中痕量基因毒性杂质2、6、12的UHPLC-Orbitrap HRMS检测方法,并进行了详尽的方法学验证。本方法灵敏度高,专属性好,回收率高,线性范围宽,填补了该领域的研究空白,可为药企对硝苯地平的质量控制提供参考,并为药监部门的监管提供有力的技术支持。
